# Integrated post-GWAS, single-cell, and functional analyses prioritize *TCTN2* at an osteoarthritis locus associated with innate immunity-related cartilage remodeling

**DOI:** 10.3389/fimmu.2026.1883412

**Published:** 2026-07-06

**Authors:** Changchun Lu, Lei Wang, Xinqi Cao, Kun Zhao, Nuo Yin, Dong Zhang

**Affiliations:** 1 Department of Orthopedics, Shanghai Jiao Tong University Affiliated Sixth People’s Hospital South Campus, Shanghai, China; 2 Department of Nuclear Medicine, Shanghai Jiao Tong University Affiliated Sixth People’s Hospital South Campus, Shanghai, China

**Keywords:** chondrocyte hypertrophy, innate immunity, osteoarthritis, single-cell RNA sequencing, *TCTN2*

## Abstract

**Background:**

Post-genome-wide association study (GWAS) interpretation of osteoarthritis (OA) increasingly requires integration with single-cell biology because many risk loci likely act through sterile inflammatory remodeling, regulatory and inflammatory chondrocyte states, and tissue-stress biology rather than through large disease-stage expression shifts. We therefore asked whether a published Transformer-based post-GWAS prioritization strategy could refine a multi-gene chr12 susceptibility locus into a tractable *TCTN2*-centered effector-gene hypothesis.

**Methods:**

Using combined hip and knee osteoarthritis summary statistics (213,839 cases and 1,080,481 controls), we built a transfer-learning variant-prioritization layer that scored 20,651,210 variants with 21 engineered features, combined these with LDSC, positional MAGMA, fibroblast and muscle eMAGMA, six-tissue SMR, and fine-mapping, and then integrated the prioritized locus with a 56,000-cell chondrocyte atlas, independent OA cartilage expression resources, OA primary-tissue eQTL maps, and ATDC5 perturbation experiments.

**Results:**

The prioritization model, used solely as a ranking layer, assigned a high prioritization score (≥ 0.99) to 17,150 variants, including the chr12 lead rs11611450 (prioritization score 0.999853), and highlighted 6,275 additional non-genome-wide-significant high-priority variants. Positional MAGMA nominated eight Bonferroni-significant genes, and eMAGMA nominated four genes in skeletal muscle and seven in fibroblasts. Within the rs11611450-linked five-gene cluster, *TCTN2* showed the strongest cross-layer convergence, with SMR support in five of six tissues, fibroblast eMAGMA support (P = 8.34 × 10^-10^), hypertrophic-chondrocyte enrichment (z = 2.20), and localization to regulatory and inflammatory chondrocyte states. Independent validation showed modestly higher *TCTN2* abundance in OA than in normal cartilage (delta = 0.45, P = 2.8 × 10^-2^), no high-grade-versus-low-grade cartilage shift (logFC = 0.053, FDR = 1.0), and strong OA-cartilage eQTL support in both low-grade and high-grade cartilage (q = 4.86 × 10–^4^ and 1.13 × 10^-2^). In ATDC5 cells, *Tctn2* knockdown reduced expression to 0.21-fold of Mock and increased *Col10a1*, *Mmp13*, and *Runx2* to 2.71-, 2.98-, and 3.53-fold, respectively, whereas overexpression and rescue reversed the same markers.

**Conclusions:**

This staged analysis nominates *TCTN2* as a plausible candidate within an unresolved chr12 osteoarthritis locus; ATDC5 perturbation indicates that *TCTN2* can modulate hypertrophic markers. Because the human-tissue association is directionally discordant, we present a locus-to-cell-state hypothesis rather than a complete genetics-to-mechanism chain, with cross-sectional shifts remaining modest and context-dependent.

## Introduction

Osteoarthritis (OA) is a whole-joint disease characterized by cartilage degeneration, synovial inflammation, and subchondral remodeling, and it carries a substantial inherited component (1–4).

Osteoarthritis is highly heritable, yet the functional interpretation of common-variant signals has lagged behind the rapid expansion of association studies. Large-scale osteoarthritis genome-wide association studies (GWAS) have expanded the field from a handful of loci to increasingly dense maps of risk, but many regions still contain several biologically plausible genes and limited experimental resolution. The largest cross-population osteoarthritis meta-analysis to date catalogued roughly 100 risk variants and prioritized effector genes across the genome, providing the reference framework against which new candidate loci are now assessed (5, 6, 41).

That translation problem is especially acute for loci that collapse onto short stretches of linkage disequilibrium containing several plausible genes and predominantly non-coding variants. In that setting, the central question is no longer whether a locus is associated with disease, but which gene, cell state, and regulatory context should be carried forward into functional follow-up.

Recent Transformer-based post-GWAS prioritization studies have suggested that learned variant-ranking models can complement conventional association testing when they are used to re-rank subthreshold signals rather than replace statistical inference (7, 8).

Single-cell studies have also changed the way osteoarthritis cartilage is interpreted. Rather than a single degenerative chondrocyte state, osteoarthritic cartilage contains homeostatic, regulatory, prehypertrophic, hypertrophic, inflammatory, and surface-associated subpopulations whose relative programs shift across disease and tissue contexts (9–11).

Primary cilia provide a plausible bridge between inherited susceptibility and chondrocyte state transitions. Transition-zone proteins organize ciliary membrane composition, and cilium-dependent signaling has been implicated in cartilage development, mechanosensing, and osteoarthritis-associated hypertrophic remodeling (12–14).

Structured post-GWAS studies in rheumatic and other complex diseases have likewise shown that staged integration can reduce diffuse association signals to a smaller set of experimentally tractable hypotheses (15–21).

Here, we asked whether a multi-gene osteoarthritis locus on chr12 could be resolved into a testable effector-gene hypothesis, whether single-cell context would support that candidate in human chondrocytes, and whether targeted perturbation would move hypertrophic marker programs in a disease-consistent direction. To do this, we treated the Transformer-based prioritization model as a ranking layer rather than a standalone claim of discovery and then asked which candidate survived convergence across post-GWAS, cell-state, and functional evidence.

## Materials and methods

### Phenotype anchoring and study design

The primary analytic anchor was combined hip/knee osteoarthritis (HIPKNEE), because it provided the most stable balance between case count and anatomical specificity among the available phenotype definitions. We compared HIPKNEE with knee OA, hip OA, and all-site OA across the first 2 million shared variants after harmonization and summarized effect-size correlation together with sign concordance before carrying any locus into downstream interpretation.

The study was organized as a serial evidence ladder: phenotype anchoring, single-nucleotide polymorphism (SNP) heritability screening, Transformer-based variant prioritization, conventional post-GWAS refinement, external OA validation, single-cell localization, and targeted functional follow-up. At each stage, downstream interpretation was carried forward only when it remained consistent with the upstream statistical anchor.

### GWAS and post-GWAS inputs

We analyzed harmonized HIPKNEE summary statistics (213,839 cases and 1,080,481 controls) as the primary discovery anchor. Both rsID-harmonized HIPKNEE statistics for locus-level analyses and a harmonized all-site OA meta-analysis table for full-variant inference were retained so that the transfer-learning and conventional post-GWAS layers were generated from the same variant universe.

Fine-mapping results were used as locus-level comparators rather than as the primary basis for candidate selection, because only 1 of 24 attempted loci achieved interpretable credible-set resolution under the available reference linkage disequilibrium (LD) setting (22).

### SNP heritability and source-task feasibility

Linkage disequilibrium score regression (LDSC) was run on rsID-pruned variant sets to estimate observed-scale SNP heritability for HIPKNEE OA and the major depressive disorder (MDD) source summary statistics, and to determine whether the source trait should be interpreted biologically or used only for representation learning (23).

Because the transfer-learning source trait was not intended as a primary etiologic comparator, cross-trait output from this step was used only to judge training feasibility and the boundary between representation learning and biological inference.

### Transformer-based variant prioritization

We adapted the published InsightGWAS strategy as a Transformer-based post-GWAS prioritization layer for osteoarthritis summary statistics (7, 8). Each variant was represented with 21 engineered features spanning association strength, allele frequency, quantitative trait locus (QTL) overlap, chromatin accessibility, transcription-factor footprints, ENCODE-style regulatory annotations, TargetScan support, segmental duplication, and LD proxy context. Source transfer-learning inputs were generated from the harmonized MDD summary statistics, and the final source matrix contained 26,851 variants (2,441 positive and 24,410 negative labels). Following the published InsightGWAS labeling convention, positive labels corresponded to association-significant and known trait-associated variants, and negative labels were sampled from the much larger pool of sub-threshold variants (here at the approximately 1:10 positive-to-negative ratio shown above). Because the labels are therefore derived from association significance, and association strength is itself one of the 21 input features, the learned score is correlated with the underlying GWAS signal by construction and is not independent of it.

Target fine-tuning used the osteoarthritis matrix with 119,625 labeled variants (10,875 positive and 108,750 negative labels), and full inference was then run across 20,651,210 all-site OA variants to generate a prioritization probability for each site. We prespecified the extreme upper tail of this distribution as a screening layer, recorded the probability tails at ≥0.90, ≥0.95, and ≥0.99, and carried the upper tail forward into downstream locus, gene, and single-cell prioritization. The model outputs were therefore used only as an exploratory tool to rank and compress the sub-threshold variant space, not as a substitute for association statistics and not as a calibrated estimate of causal or pathogenic probability. Model fitting followed the published InsightGWAS training protocol, with an internal training/validation split used during fine-tuning. Because the prioritization output is by construction correlated with association strength and linkage-disequilibrium structure, we did not treat it as evidence independent of the GWAS and did not rely on held-out discrimination or calibration metrics; every locus and gene carried forward was instead required to be supported by orthogonal SMR, eMAGMA, single-cell, and external expression or eQTL evidence.

### Gene prioritization layers

Positional Multi-marker Analysis of GenoMic Annotation (MAGMA) was used to nominate locus-proximal gene-level signals, and tissue-sensitive expression-informed MAGMA (eMAGMA) results from muscle-skeletal and fibroblast eQTL references were used to identify expression-informed gene burden. Gene-set testing was run in parallel to determine whether the signal concentrated into broader pathway themes. Summary-data-based Mendelian randomization (SMR) was summarized across GTEx v8 Binary eQTL Summary Data (BESD) resources from skeletal muscle tissue, cultured fibroblasts, whole blood, subcutaneous adipose tissue, tibial nerve, and sun-exposed skin. We interpreted SMR as expression-linked prioritization, not as direct proof of shared causal mediation. The eMAGMA and SMR tissue panels were drawn from the GTEx eQTL references with adequate instrument power and did not include cartilage, which is not represented in GTEx; the gene-level expression evidence is therefore from non-cartilage proxy tissues, with cartilage-relevant regulation entering only later through the rescued OA primary-tissue eQTL layer. For the chr12-cluster probes, the SMR HEIDI heterogeneity test could not be evaluated because the cis instrument was reduced to the single lead variant rs11611450 (HEIDI requires multiple cis-SNPs), so SMR at these probes cannot, by itself, separate a shared causal variant from linkage (24–27).

The model-derived probabilities were not treated as a replacement for these conventional layers. Instead, they were used to ask whether the learned variant-prioritization surface converged on the same loci and genes that were supported by expression-linked and locus-level analyses. Candidate integration was summarized with a convergence score, defined as the number of independent evidence layers a gene satisfied (maximum of six): genome-wide-significant GWAS support (P < 5 × 10^-8^); Bonferroni-significant SMR in at least one of the six tissues; Bonferroni-significant fibroblast eMAGMA; hypertrophic-chondrocyte single-cell enrichment (z ≥ 1); any cell-type-specific single-cell signal (|z| ≥ 1.5); and cilium-marker co-expression in hypertrophic chondrocytes. Each satisfied layer contributed one point, and the score was used solely to order candidates for the wet-lab nomination step, not as a formal statistical test.

### Independent OA expression and OA primary-tissue eQTL validation

To add orthogonal human-tissue support without new wet-lab work, we analyzed processed bulk-cartilage expression matrices from GSE114007 and compared OA versus normal cartilage on a per-gene basis with Welch t-tests using the parsed sample columns distributed in the public download (20 normal and 22 osteoarthritic cartilage samples, n = 42) (28). We also computed Spearman correlations between *TCTN2* and prespecified hypertrophy-associated markers across all GSE114007 samples (n = 42) and within OA cartilage only (n = 22).

We summarized a 2024 MSK-KP primary-cartilage differential-expression workbook comparing low-grade and high-grade OA cartilage, retaining the reported log fold changes and adjusted P values exactly as distributed in the public workbook. Because the workbook defined low-grade cartilage as the reference, positive values were interpreted as relatively higher expression in high-grade cartilage.

To strengthen the regulation layer, we rescued OA primary-tissue all-pairs eQTL resources for low-grade cartilage, high-grade cartilage, and synovium from the public Steinberg 2021 OA molecular-QTL release and extracted rows for the chr12 candidate genes plus the *GLIS3* comparator locus (29). We summarized nominal association statistics, permutation-derived q values, top variants, and eGene flags by gene and tissue. Because this extension used the coordinate identifiers present in the rescued files rather than a fully harmonized lead-variant rsID colocalization workflow, the resulting OA tissue eQTL layer was interpreted as cartilage-relevant regulation support rather than as formal colocalization evidence.

### Single-cell analysis

Public chondrocyte single-cell datasets were integrated with Seurat reciprocal principal-component analysis (RPCA) and projected onto a reference taxonomy derived from GSE104782, yielding an atlas of approximately 56,000 cells from three healthy and four osteoarthritic donors and nine retained chondrocyte states (9–11, 30).

We used AUCell gene-set activity scoring and a single-cell disease-relevance score (scDRS)-style enrichment summary to score osteoarthritis GWAS-derived and chr12-locus gene signatures, single-cell regulatory network inference and clustering (SCENIC) to summarize regulon structure, Slingshot to model cell-state progression from homeostatic toward hypertrophic branches, Milo to assess differential abundance, and CellChat plus NicheNet to summarize differential communication and ligand programs (31–36).

Public datasets were integrated and batch-corrected with Seurat reciprocal-PCA, and the donor was treated as the biological replicate unit. Because only seven donors were available and the integrated atlas combined public datasets that differ in batch, anatomical site, dissociation protocol, and disease severity, condition-level single-cell results are reported as hypothesis-generating: cell-state polygenic enrichment (scDRS, assessed against a matched control-gene Monte-Carlo null at the cell level) and graph-based differential abundance (Milo, which models per-donor counts) were prioritized over donor-level abundance contrasts, and confirmation with donor-level pseudobulk aggregation or generalized linear mixed models carrying a per-donor random effect was identified as the appropriate next step (39, 40).

### ATDC5 functional follow-up

ATDC5 cells were used as a chondrogenic differentiation model because they provide a reproducible hypertrophy-prone system for short-turnaround perturbation assays (37). Five groups were analyzed: Mock, siNC, si-*Tctn2*, oe-*Tctn2*, and Rescue. Relative mRNA levels for *Tctn2*, *Col10a1*, *Mmp13*, and *Runx2* were quantified by the 2^-ΔΔCt^ method with *Gapdh* as the internal control. Western blotting assessed TCTN2 and COL10A1 directionally, and all experiments were summarized across three biological replicates per condition.

### Statistics

For the ATDC5 assay, one-way ANOVA was used to compare the five groups for each quantitative polymerase chain reaction (qPCR) readout, followed by Tukey’s post-hoc tests. The manuscript reports group means from three biological replicates per condition. Single-cell differential expression was interpreted conservatively because donor-level power was modest (three healthy and four osteoarthritic donors), and pathway or cell-state summaries were emphasized when they provided more stable readouts. Empirical permutation P values are reported as P < 1/(N + 1) when they reached the resolution limit of the permutation null rather than as exactly 0, and the permutation unit (cell level for the scDRS-style module-score enrichment, with 30 random gene-set permutations; per-donor counts for Milo) is stated with each result. Between-condition single-cell findings are presented as hypothesis-generating. All reported tests were two-sided, and multiple-testing-adjusted statistics were used where applicable.

## Results

### Combined hip/knee osteoarthritis provided the most stable anchor across related phenotype definitions

Among the available osteoarthritis phenotype definitions, combined hip/knee OA showed the strongest concordance with knee OA, with an effect-size correlation of 0.93 across the first 2 million shared variants and a same-direction rate of 0.86. Concordance was more moderate for all-site OA (beta correlation 0.66; same-direction rate 0.77) and hip OA (beta correlation 0.54; same-direction rate 0.72).

These comparisons supported the use of combined hip/knee OA as the primary discovery anchor for locus interpretation. They also indicated that the strongest downstream signals were most likely to reflect shared knee-dominant osteoarthritis architecture rather than isolated phenotype-specific noise.

### Heritability screening supported the use of the source trait for representation learning rather than biological inference

Observed-scale SNP heritability for combined hip/knee OA was 0.022 ± 0.0074, whereas the MDD source summary statistics showed observed-scale SNP heritability of 0.0554 ± 0.0043.

A direct OA–MDD genetic correlation estimate did not yield a stable result because the LD Score regression run failed due to insufficient overlap (failed_insufficient_overlap). We therefore retained MDD strictly as a source task for representation learning and did not interpret it as part of the osteoarthritis biological results. MDD was chosen as the transfer-learning source following the published InsightGWAS precedent, because its large, well-powered, and feature-complete summary statistics provide a stable pretraining signal, not because of any biological proximity to osteoarthritis. The learned prioritization surface is agnostic to source-trait biology, and the failed OA–MDD genetic correlation estimate should not be interpreted as implying any biological relationship between the two conditions.

### A transformer-based prioritization layer compressed the variant space while preserving the statistical anchor

The Transformer-based prioritization stage scored 20,651,210 variants with the 21-feature transfer-learning model. A total of 17,150 variants reached a prioritization score ≥0.99 and 17,665 reached a score ≥0.95. The chr12 lead variant rs11611450 itself received a score of 0.999853 (these are model ranking scores, not calibrated pathogenicity probabilities). Because positive labels were defined from association significance, and association strength is one of the input features, these scores are correlated with the underlying GWAS signal by construction. The concordance at rs11611450 therefore reflects internal consistency of the ranking layer rather than independent statistical confirmation, and we used the score only to compress and rank the sub-threshold variant space.

The prioritization model also expanded the tractable search space beyond conventional genome-wide significance. In addition to retaining all known genome-wide significant signals, the probability ≥0.99 tier contained 6,275 variants with P_meta ≥ 5 × 10^-8^, providing a focused non-genome-wide-significant pool for downstream filtering. This allowed us to preserve the conventional statistical anchor while reducing the long subthreshold tail to a subset that could realistically be carried into gene-level and cell-state analyses. We emphasize that the chr12 nomination did not depend on this layer’s distinctive output: rs11611450 is genome-wide significant and would have been carried forward by conventional analysis, and none of the 6,275 sub-threshold high-priority variants entered the chr12 or *TCTN2* story. Therefore, the prioritization layer served as a variant-space compression and ranking step, and the candidate nomination rests on the convergence of conventional post-GWAS, single-cell, and functional evidence rather than on the model.

### Human osteoarthritis chondrocyte atlasing localized the genetic signal to prehypertrophic and hypertrophic contexts

**Figure 1:**
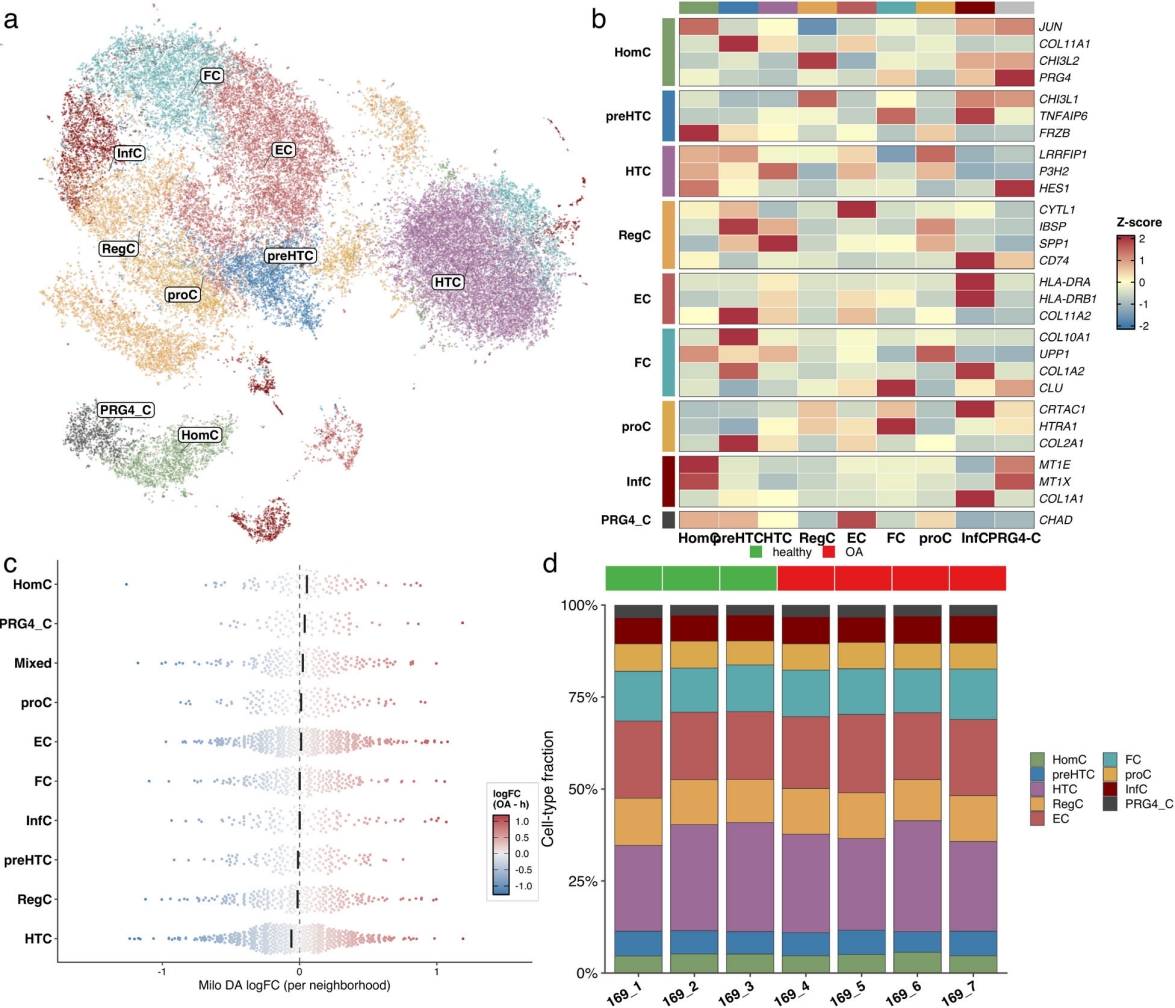
Single-cell atlas of human osteoarthritis chondrocytes. **(A)** Integrated chondrocyte UMAP showing nine annotated states across healthy and osteoarthritic donors. **(B)** Canonical marker heatmap used for state annotation. **(C)** Milo differential-abundance summary showing that donor-level shifts were weak. **(D)** Donor-level cell-state composition across the included samples.

The single-cell phase integrated approximately 56,000 chondrocytes from three healthy and four osteoarthritic donors into a nine-state atlas spanning homeostatic, regulatory, effector, fibrocartilage, proliferative, inflammatory, superficial-zone, prehypertrophic, and hypertrophic populations. Donor-level differential abundance was weak, with 0 Milo neighborhoods reaching FDR < 0.05, suggesting, as a hypothesis to be confirmed in larger donor panels, that the osteoarthritis signal was better explained by state-level remodeling than by large shifts in subtype presence. The scDRS-style enrichment score was nominally highest in prehypertrophic chondrocytes (mean 0.0066, n = 4,743 cells), with closely similar values in homeostatic (0.0058, n = 7,270) and regulatory (0.0052, n = 43,987) chondrocytes. The three retained states therefore spanned a narrow range (0.0052–0.0066), and the rank ordering should be interpreted cautiously. The empirical permutation P values for all three retained states reached the resolution limit of the permutation null and are therefore reported as P < 1/(N + 1) with N = 30 random gene-set permutations (i.e., P < 0.03), rather than as exactly 0. These are cell-level permutation P values from a Seurat module-score, scDRS-style test rather than a donor-level test, and the coarse 30-permutation null limits their precision (**Figure 1**).

**Figure 2:**
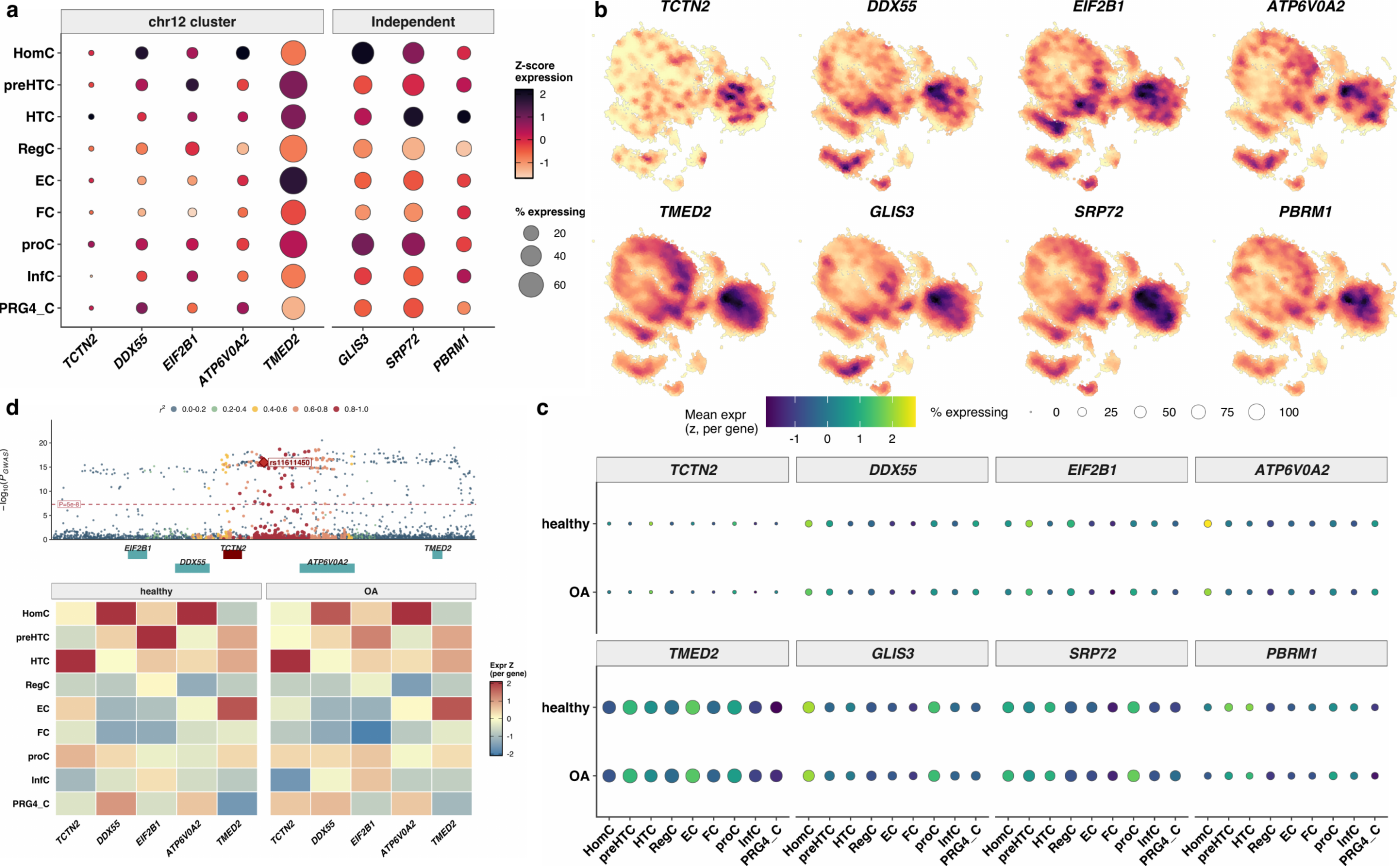
Cell-state localization of the chr12 candidate cluster and comparator loci. **(A)** State-level dot plot for chr12-cluster genes and independent comparator genes. **(B)** Per-gene UMAP density maps for the same candidate set. **(C)** Fraction of expressing cells (dot size) and mean expression (color, z-scored per gene) for the chr12-cluster and comparator genes across chondrocyte states in healthy versus osteoarthritic cartilage. **(D)** Local view of the rs11611450 locus together with matched state-level expression heatmaps for the chr12-cluster genes.

Candidate genes from the chr12 locus were detectable in chondrocytes, but their osteoarthritis-versus-healthy abundance shifts were modest. None of the 27 candidate-gene comparisons retained significance after multiple-testing correction, and the candidate panel showed uniformly small effect sizes. In contrast, state-level enrichment was sharper: prehypertrophic chondrocytes showed the highest OA-GWAS enrichment score (mean 0.0066), and the *TCTN2*-centered integrated evidence matrix showed its strongest gene-level single-cell signal in hypertrophic chondrocytes (z = 2.20; **Figure 2**). This dissociation between modest steady-state abundance change and stronger cell-state localization suggested that the inherited signal acts more through altered differentiation context than through large cross-sectional expression shifts.

### Trajectory and communication analyses favored a regulatory interpretation over large on-off expression shifts

**Figure 3:**
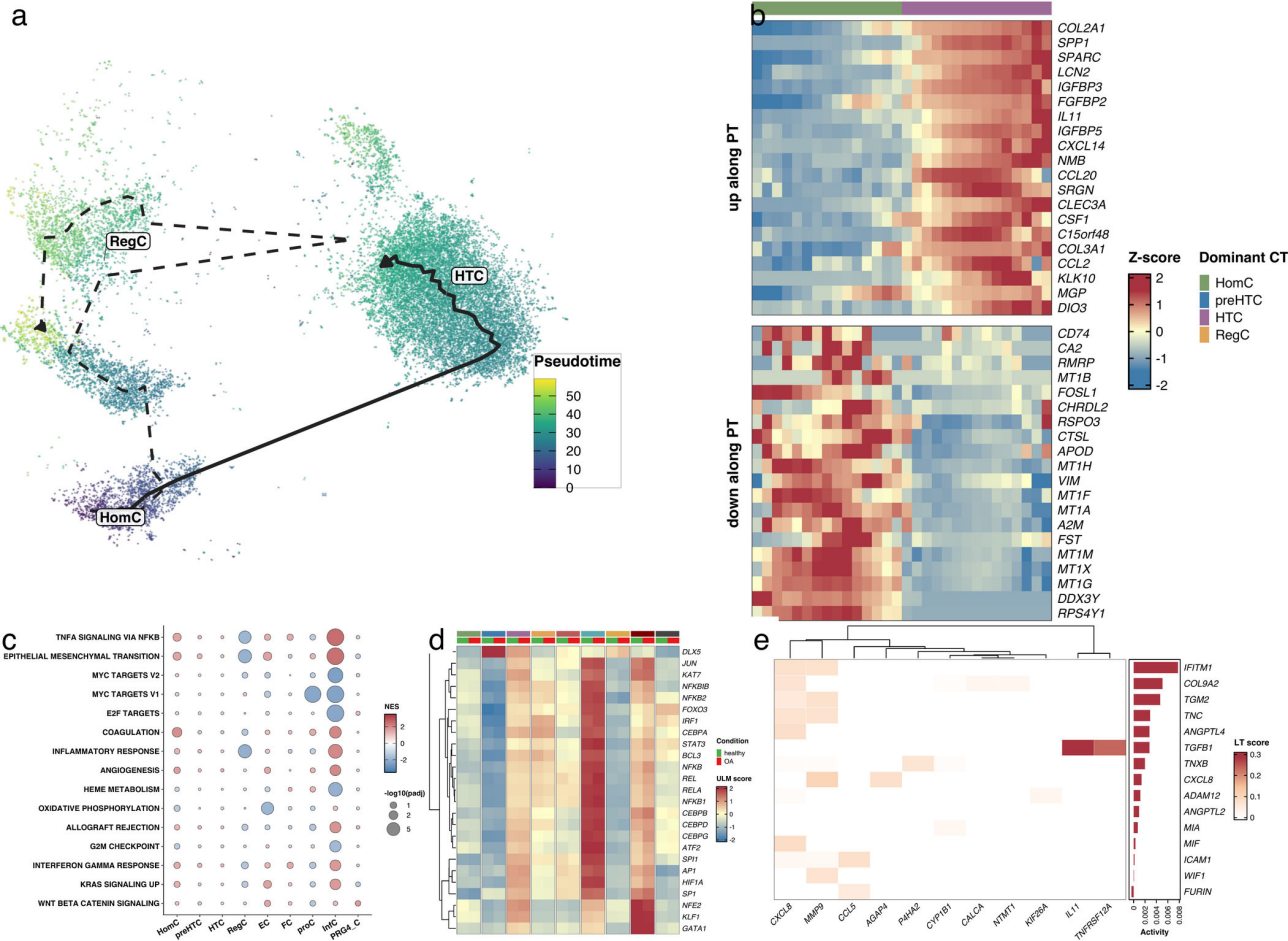
Trajectory, pathway, and regulator context of osteoarthritis chondrocyte progression. **(A)** Slingshot trajectory projected onto the integrated atlas, showing the homeostatic-to-prehypertrophic-to-hypertrophic branch. **(B)** Pseudotime-associated gene modules stratified by dominant cell state. **(C)** Pathway-enrichment shifts across major chondrocyte states. **(D)** Regulator-activity heatmap comparing healthy and osteoarthritic chondrocytes. **(E)** Ligand–target activity map aligned with the hypertrophic transition.

**Figure 4:**
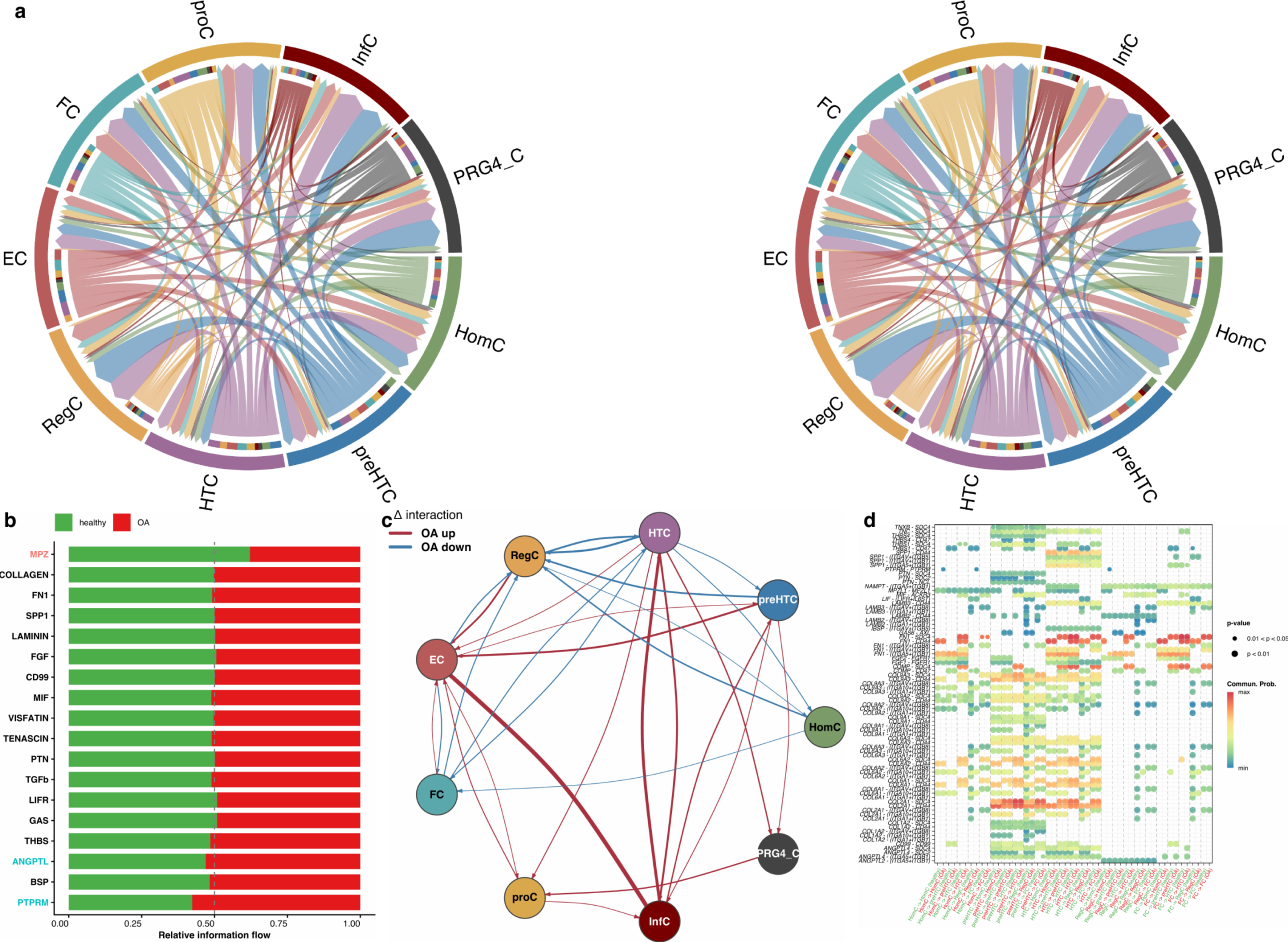
Differential cell–cell communication in osteoarthritis cartilage. **(A)** Global communication networks in healthy (left) and osteoarthritic (right) cartilage. **(B)** Relative information flow for pathways that differed between conditions. **(C)** Differential sender–receiver network across chondrocyte states. **(D)** Ligand–receptor and pathway interaction map underlying the remodeling signal.

Slingshot trajectory analysis resolved a homeostatic-to-prehypertrophic-to-hypertrophic branch as the dominant osteoarthritis-relevant progression path. Pathway and regulon overlays placed matrix-remodeling, inflammatory, and differentiation programs along that branch, with SPP1, LCN2, IL11, and CCL20 among the genes increasing along pseudotime, while CellChat and NicheNet highlighted TGFβ-, collagen-, and inflammatory ligand structures as the dominant communication backdrop of the hypertrophic transition (**Figures 3**, **4**).

Together, these analyses suggested that the inherited signal was more consistent with altered baseline regulatory tone or transition propensity than with strong disease-stage induction of the candidate genes themselves. That interpretation fit the chr12 locus particularly well, because the locus retained strong expression-linked support across tissues while its candidate genes showed only modest OA-versus-healthy abundance shifts in cartilage. In other words, the genetic signal aligned more with where chondrocytes were moving in state space than with a single on-off expression contrast.

### Conventional post-GWAS layers bounded the candidate space and converged on *TCTN2* within the rs11611450 cluster

**Figure 5:**
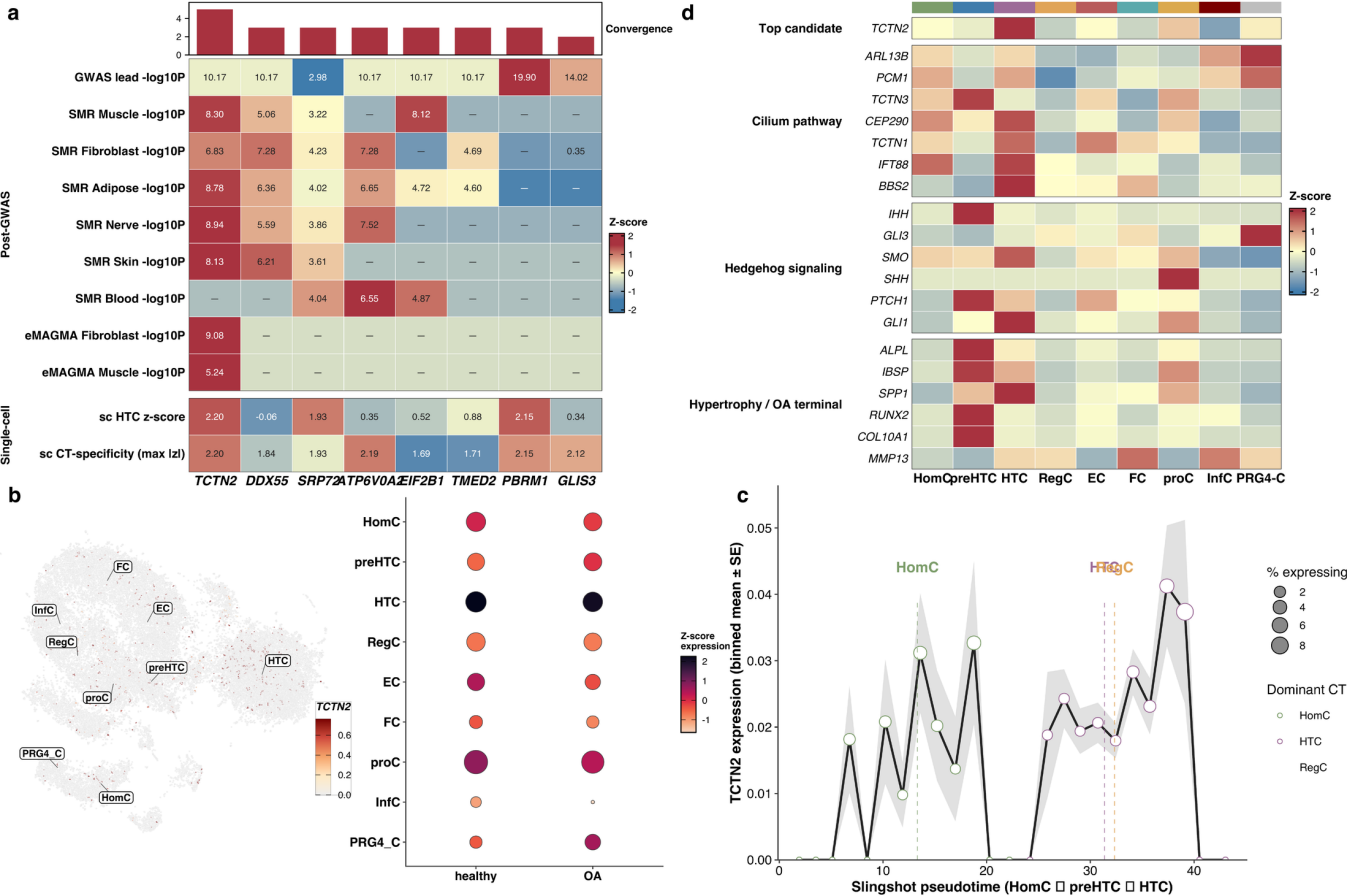
Cross-layer prioritization selected *TCTN2* for functional follow-up. **(A)** Convergence matrix across GWAS strength, SMR breadth, eMAGMA support, and single-cell context. **(B)** *TCTN2* localization across chondrocyte states, shown as UMAP signal and healthy-versus-osteoarthritis state summary. **(C)** *TCTN2* expression along the homeostatic-to-hypertrophic pseudotime branch. **(D)** State- and pathway-resolved heatmap for *TCTN2* and comparator genes across cilium, Hedgehog, and hypertrophy-related programs.

Conventional post-GWAS analyses did not reduce the study to a single obvious effector gene, but they did define a bounded candidate space (**Figure 5**). Positional MAGMA identified eight Bonferroni-significant genes, including *PBRM1* (P = 4.45 × 10^-14^). eMAGMA identified four significant genes in skeletal muscle tissue and seven in fibroblasts, with three shared between tissues. In this setting, *TCTN2* remained significant in fibroblasts (P = 8.34 × 10^-10^) but not in skeletal muscle tissue, indicating tissue-sensitive support rather than uniform expression-linked signal across all references.

Pathway-level signal was weaker and more diffuse than gene-level signal in both positional MAGMA and eMAGMA analyses, without a single dominant enrichment pattern. This suggested that the study was more informative at the level of bounded candidate genes than at the level of a unifying pathway theme.

The strongest osteoarthritis signal carried forward into downstream integration was the chr12 lead variant rs11611450 (P = 6.71 × 10^-11^), which mapped to a compact five-gene cluster containing *TCTN2*, *DDX55*, *EIF2B1*, *ATP6V0A2*, and *TMED2*. Cross-tissue SMR showed that the locus had broad expression-linked architecture, with Bonferroni support for *TCTN2* in five tissues, *DDX55* in five, *ATP6V0A2* in four, *EIF2B1* in three, and *TMED2* in two. Fibroblast eMAGMA added independent support for *TCTN2* (P = 8.34 × 10^-10^), while *GLIS3* remained the strongest independent positional and fine-mapped comparator locus.

Fine-mapping provided an instructive contrast. Of 24 attempted lead loci, only 1 yielded a credible-set solution under the available EUR LD proxy, and that successful example was an independent GLIS3 locus (chr9_4291928_GLIS3), in which rs12350099 showed near-unit posterior inclusion probability (PIP = 0.9986). The chr12 cluster was therefore not nominated because it was the most resolved fine-mapping signal; it was nominated because it remained the most coherent expression-linked and single-cell-supported unresolved locus.

A broader cross-candidate comparison showed that some genes, such as *SRP72*, carried wide SMR breadth from weaker independent loci. We therefore prioritized the final wet-lab candidate by joint evidence rather than by any single metric. Within the chr12 cluster, *TCTN2* had the highest integrated convergence score (5), whereas neighboring genes remained at 3 or lower. *TCTN2* led the cluster specifically because it was the only chr12 gene to add Bonferroni-significant fibroblast eMAGMA support and hypertrophic-chondrocyte single-cell enrichment (z = 2.20) on top of the genome-wide-significant GWAS and 5-of-6-tissue SMR breadth it shared with *DDX55. DDX55* scored 3 because it lacked both the eMAGMA and hypertrophic-chondrocyte single-cell layers. That ranking gave *TCTN2* the clearest biologic rationale for follow-up because its genetic anchoring, expression-linked support, and cilium-related interpretation were coherent across layers.

### *Tctn2* perturbation shifted hypertrophic marker programs in ATDC5 cells

**Figure 6:**
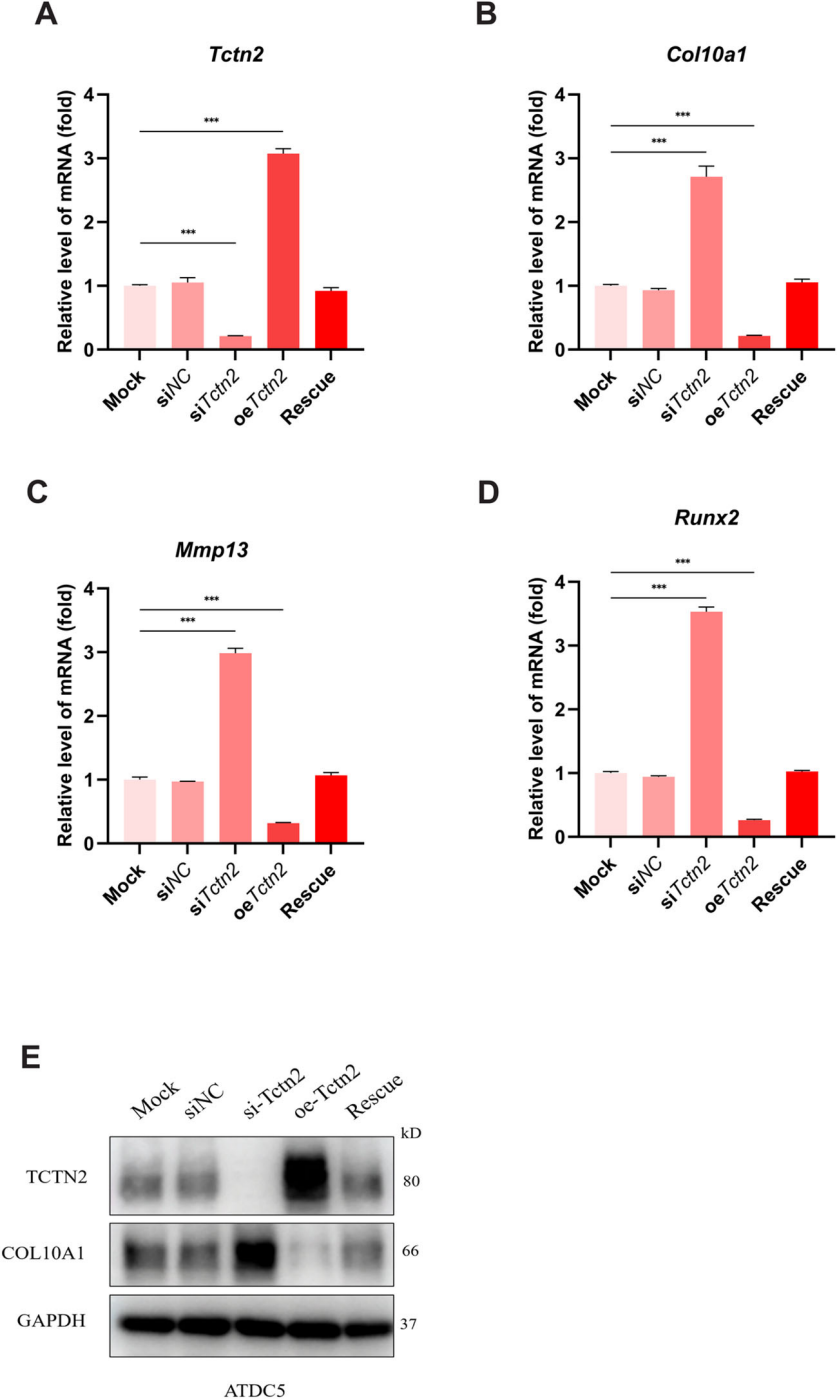
ATDC5 functional follow-up of *Tctn2*. **(A–D)** qPCR quantification of *Tctn2*, *Col10a1*, *Mmp13*, and *Runx2* across Mock, siNC, si-*Tctn2*, oe-*Tctn2*, and Rescue groups. **(E)** Representative western blots for TCTN2 and COL10A1 with GAPDH as the loading control. Knockdown promoted a hypertrophic marker program, whereas overexpression and rescue shifted the readouts back toward baseline. Statistical comparisons by one-way ANOVA with Tukey's post hoc test; ***P < 0.001.

Functional follow-up supported the direction suggested by the human genetics and single-cell analyses (**Figure 6**). Across the five-group ATDC5 design, all four qPCR readouts differed significantly by one-way ANOVA (for *Tctn2*, P = 2.69 × 10^-13^; for *Col10a1*, P = 6.12 × 10^-11^; for *Mmp13*, P = 6.37 × 10^-14^; and for *Runx2*, P = 1.31 × 10^-15^). Knockdown reduced *Tctn2* to 0.21-fold of Mock, whereas overexpression increased it to 3.07-fold, and Rescue returned it to 0.92-fold.

Loss of *Tctn2* was accompanied by a consistent hypertrophic signature. Relative to Mock, *Col10a1*, *Mmp13*, and *Runx2* rose to 2.71-, 2.98-, and 3.53-fold, respectively, whereas overexpression suppressed the same markers to 0.21-, 0.32-, and 0.26-fold. Mock and siNC were not detectably different across the four readouts, whereas Rescue returned all three markers close to baseline (1.06, 1.07, and 1.02, respectively), with Mock-versus-Rescue Tukey P values of 0.912, 0.366, and 0.936; the disease-relevant contrasts were each strongly significant, with siNC versus si-Tctn2, si-Tctn2 versus Rescue, and siNC versus oe-Tctn2 all giving P_adj < 0.0001 (Tukey HSD) for Col10a1, Mmp13, and Runx2. Western blotting showed concordant directional changes in TCTN2 and COL10A1, consistent with a role for *TCTN2* in restraining the hypertrophic marker program in this *in vitro* model; this direction is, however, not aligned with the human-tissue expression data (see Discussion).

### Independent OA expression and OA primary-tissue eQTL rescue refined the chr12 interpretation

**Figure 7:**
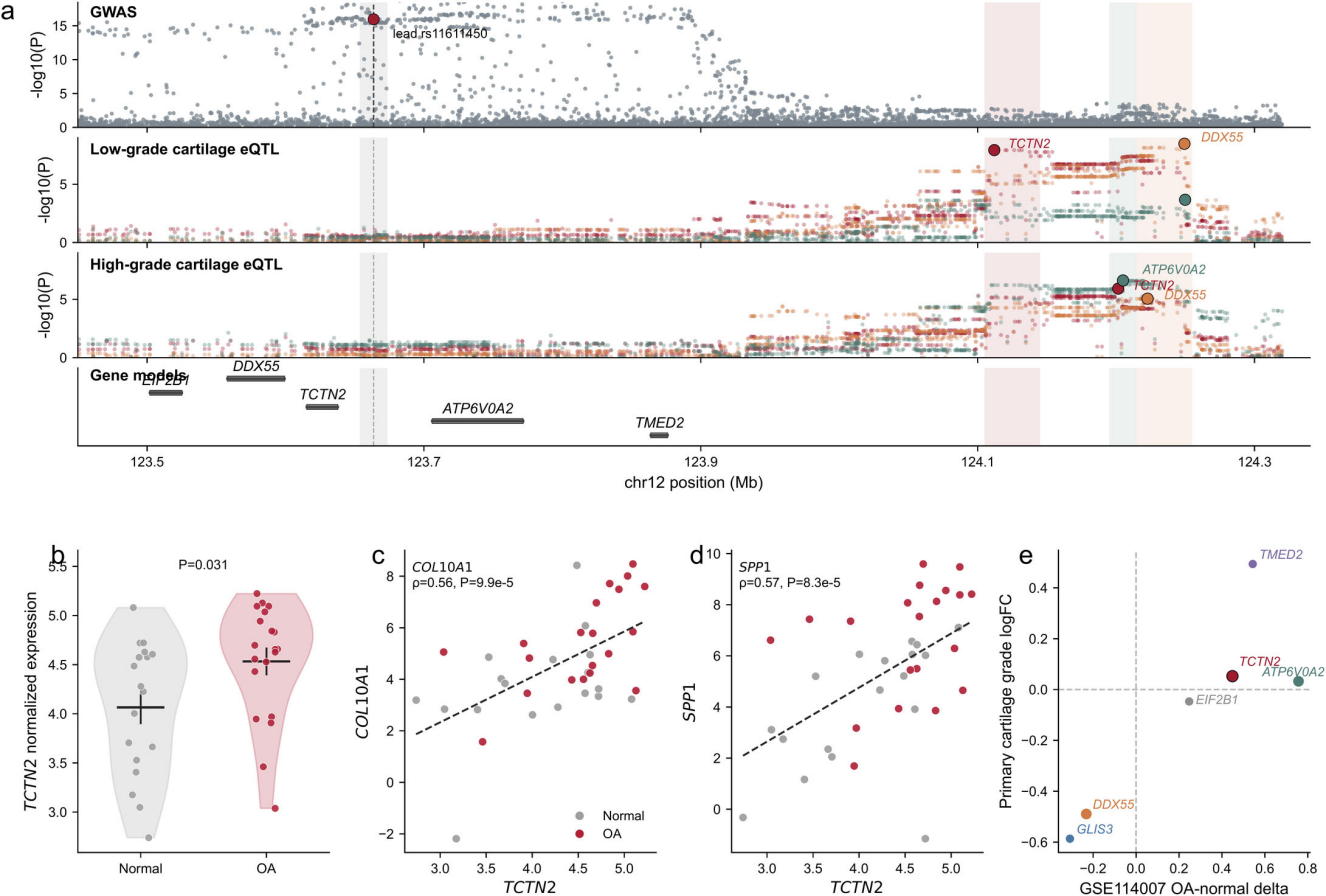
Locus-centric external validation of the chr12 osteoarthritis candidate region. **(A)** Shared-coordinate chr12 view aligning the OA GWAS peak, rescued low-grade cartilage eQTL signal, rescued high-grade cartilage eQTL signal, and local gene models across the *TCTN2–DDX55* interval. The rs11611450 lead association and recovered eQTL hotspots are shown in the same genomic window. **(B)** GSE114007 sample-level *TCTN2* distribution in normal and osteoarthritic cartilage. **(C)** Sample-level association between *TCTN2* and *COL10A1* across GSE114007 cartilage samples. **(D)** Sample-level association between *TCTN2* and *SPP1* across the same dataset. **(E)** Cross-cohort concordance comparing GSE114007 OA–normal expression shifts with 2024 primary-cartilage high-grade–versus–low-grade shifts for chr12 candidates and the *GLIS3* comparator.

A shared-coordinate view of the chr12 interval clarified the regulatory architecture of the locus (**Figure 7**). The OA association peak remained centered on rs11611450, whereas the rescued cartilage eQTL hotspots occupied the distal part of the same window rather than collapsing onto a directly recovered manuscript lead-rsID match. Within that frame, *TCTN2* emerged as an expression-QTL gene (eGene) in low-grade cartilage (q = 4.86 × 10^-4^) and high-grade cartilage (q = 1.13 × 10^-2^), with the strongest low-grade signal centered on 12:124111999, whereas synovium support was weaker (q = 2.73 × 10^-1^). *DDX55* also showed cartilage eGene support (low-grade q = 1.67 × 10^-4^; high-grade q = 4.44 × 10^-2^), but *GLIS3* did not reach eGene status in the rescued OA tissue map, and its best recovered tissue-level q value remained 2.3 × 10^-1^. Because the exact manuscript lead rsIDs were not directly recovered from the rescued OA tissue files, this layer was interpreted as cartilage-relevant regulation support rather than formal lead-variant colocalization.

The expression layer pointed to a remodeling context rather than a single abundance-direction model. In GSE114007, *TCTN2* was modestly higher in OA than in normal cartilage (delta, the osteoarthritis-minus-normal difference in normalized expression, 0.45; P = 2.8 × 10^-2^), and across all GSE114007 samples it correlated positively with hypertrophy-associated markers, including *COL10A1* (rho = 0.56; P = 9.9 × 10^-5^) and *SPP1* (rho = 0.57; P = 8.29 × 10^-5^). In the independent 2024 primary-cartilage high-grade-versus-low-grade comparison, however, *TCTN2* was essentially unchanged (logFC 0.053; FDR = 1.0), whereas *GLIS3* was lower (logFC -0.587; FDR = 4.15 × 10^-13^), *DDX55* was also lower (logFC -0.489; FDR = 5.37 × 10^-13^), and *TMED2* was higher (logFC 0.494; FDR = 3.69 × 10^-8^). Taken together, the two external expression cohorts and the OA-cartilage eQTL layer again favored *TCTN2* not because it was the most strongly shifted bulk-expression gene, but because it showed the most coherent combination of cartilage regulatory support and human-tissue remodeling context among the chr12 candidates.

## Discussion

By combining phenotype anchoring, post-GWAS prioritization, external human-tissue validation, single-cell localization, and targeted perturbation, this study reduces a multi-gene osteoarthritis association signal to a tractable effector-gene hypothesis centered on *TCTN2*. The key advance is not the discovery of an additional locus, but the conversion of an unresolved chr12 signal into a cilium-linked candidate that retained support across gene-prioritization, OA-cartilage regulation, cell-state localization, and perturbation assays. The data therefore support prioritization rather than a definitive mechanism, but they materially sharpen how this locus can now be interpreted and tested.

That refinement matters because recent osteoarthritis GWAS have expanded the number of implicated loci far faster than the number of loci that can be reduced to experimentally tractable genes (5, 6). Our phenotype comparisons agree with those studies in showing that osteoarthritis risk contains both shared and anatomically weighted components, and they extend that literature by showing that combined hip/knee OA tracked knee OA much more closely than hip OA or all-site OA in the present dataset. This suggests that the strongest downstream signals in this manuscript largely reflect knee-dominant shared architecture rather than diffuse phenotype heterogeneity.

The chr12 locus illustrates why larger association studies do not by themselves resolve effector genes. Fine-mapping yielded a credible-set solution at only one locus, whereas the rs11611450 region remained unresolved at the variant level despite strong association support. What narrowed that region was the agreement between the learned prioritization surface, six-tissue SMR breadth, fibroblast eMAGMA support, and the rescued OA primary-tissue eQTL layer. This is consistent with broader post-GWAS experience and with the OA molecular-QTL map from Steinberg and colleagues, which showed that cartilage and synovium can provide disease-relevant regulatory information that is not captured by proxy tissues alone (29). In the present study, that layer especially strengthened *TCTN2*, which emerged as an OA-cartilage eGene in both low-grade and high-grade cartilage, whereas the fine-mapped comparator *GLIS3* did not show the same OA tissue eGene pattern.

The rescued expression resources also argue against an overly simple abundance-based interpretation. In GSE114007, *TCTN2* was modestly higher in OA than in normal cartilage, whereas in the 2024 primary-cartilage high-grade-versus-low-grade comparison, it was essentially unchanged. By contrast, *GLIS3* and *DDX55* were reduced in advanced cartilage, and *TMED2* was increased. Public OA transcriptome studies have long shown that large differential expression signatures can reflect disease-stage remodeling and tissue composition as much as direct causal mediation (28). Our external validation fits that pattern: *TCTN2* was not the most dramatically shifted bulk-expression gene, but it was the gene with the clearest combination of cartilage regulatory support and downstream functional coherence. This distinction helps explain why the chr12 wet-lab follow-up remained focused on *TCTN2* rather than simply on the strongest cross-sectional differential expression hit.

The single-cell phase fits closely with recent osteoarthritis atlases that emphasize prehypertrophic, hypertrophic, inflammatory, and regulatory chondrocyte programs as central components of cartilage degeneration (9–11). We recovered the same broad state structure and again found that the prehypertrophic-to-hypertrophic branch is a major disease-relevant axis, but the strongest inherited signal appeared at the level of cell-state context rather than large OA-versus-healthy differences in candidate-gene abundance. This extends the atlas literature by suggesting that common-risk loci may bias the trajectory that chondrocytes take through disease space, even when the steady-state expression difference of the nominated gene itself is small. The positive cross-sample correlation between *TCTN2* and hypertrophy-associated markers in GSE114007 is, on its face, in tension with the perturbation phenotype: it indicates that *TCTN2* tracks remodeling context in human tissue even though experimental reduction of *Tctn2* worsens the hypertrophic program in the perturbation system.

*TCTN2* is a biologically plausible mediator of that effect. It encodes a transition-zone component of the primary cilium, and cilium-dependent signaling has been linked to cartilage development, Hedgehog responsiveness, and osteoarthritis-related remodeling (12–14). Our ATDC5 data were consistent with that biology: lowering *Tctn2* increased *Col10a1*, *Mmp13*, and *Runx2*, whereas overexpression and rescue shifted the same markers back toward baseline. This agrees with prior work placing RUNX2-linked hypertrophy and matrix-remodeling programs near the center of osteoarthritis progression, and it extends that literature by connecting an inherited osteoarthritis locus to a specific cilium-linked follow-up candidate. To our knowledge, the chr12 rs11611450 cluster and *TCTN2* have not previously been highlighted as an effector-gene candidate in the major osteoarthritis GWAS, and transition-zone ciliary genes have only rarely been nominated at osteoarthritis loci, which is part of what makes this candidate worth testing (6, 38, 41).

Several limitations define the current boundary of inference. The GWAS anchor is European-focused, and the chr12 locus does not yet have formal lead-variant cartilage colocalization support. The transfer-learning prioritization layer was used only to rank and compress the variant space and, because its labels derive from association significance and association strength is among its input features, it recapitulates rather than independently validates the GWAS signal and was not accompanied by held-out discrimination or calibration metrics. The major depressive disorder source trait was likewise suitable only for representation learning and not for biological comparison. The single-cell atlas combines public datasets that differ in batch, anatomical site, dissociation protocol, and disease severity, and it is still based on only seven donors; therefore, between-condition contrasts are hypothesis-generating and require donor-level pseudobulk or mixed-model confirmation. In addition, the functional follow-up used a murine ATDC5 system with marker-focused qPCR and western blot readouts rather than direct cilium imaging, Hedgehog assays, or genome editing at rs11611450. The rescued OA tissue eQTL layer materially improves the regulatory interpretation, but because the recovered files did not directly return the manuscript lead rsIDs in the current extraction route, it still cannot be presented as definitive lead-variant colocalization. Broad SMR support across GTEx tissues is useful for prioritization, but it rests on non-cartilage proxy tissues and, for the single-variant chr12 instrument, could not be filtered by the SMR HEIDI heterogeneity test (so a shared causal variant cannot be formally distinguished from linkage). It likewise does not prove that the same causal variant acts through cartilage-resident *TCTN2* regulation *in vivo*. A further central limitation is the directional inconsistency between the human and experimental data. In cartilage tissue, *TCTN2* was modestly higher in osteoarthritis and correlated positively with hypertrophic markers, whereas experimental *Tctn2* knockdown increased the same markers in ATDC5 cells. This discordance could reflect compensatory upregulation in diseased tissue, cell-composition confounding in bulk cartilage, disease-stage dependence, or intrinsic differences between the murine ATDC5 model and human cartilage, and it currently precludes a unidirectional protective interpretation of *TCTN2*; resolving it will require allele-specific and human primary-chondrocyte experiments. More specifically, the positive bulk-tissue correlation is confounded by cell-state composition and disease-stage remodeling and is not a statement of causal direction, whereas the only directional, causal readout in this study is the ATDC5 perturbation; the protective interpretation of *TCTN2* therefore rests on that experiment rather than on the cross-sectional human expression data.

Taken together, the results nominate *TCTN2* as the most coherent expression-linked candidate gene at the chr12 osteoarthritis locus and provide a focused hypothesis for the next mechanistic phase of follow-up. The most direct next steps are cartilage-relevant colocalization, allele-specific regulatory assays, and locus-aware perturbation experiments that can test whether cilium-linked modulation of chondrocyte state transition is the mechanism connecting this locus to disease risk.

## Conclusions

Integrating phenotype anchoring, post-GWAS refinement, single-cell localization, and ATDC5 follow-up prioritized *TCTN2* as the most coherent candidate gene within the unresolved chr12 osteoarthritis locus. ATDC5 perturbation indicates that *TCTN2* can modulate the hypertrophic marker program. Because experimental *Tctn2* reduction increased hypertrophic markers, whereas *TCTN2* was modestly higher in osteoarthritic cartilage and correlated positively with those markers, the directional relationship between the human-tissue signal and the perturbation phenotype is not yet resolved. We therefore present a locus-to-cell-state hypothesis rather than a complete genetics-to-mechanism chain, and note that the locus likely operates through subtle regulatory effects rather than large steady-state expression shifts.

## Data Availability

Public datasets used in this study are cited in the article and are available from the referenced sources. No new primary data were generated in this study; every dataset analyzed is publicly available and is cited in the article with its original accession or source, so the data underlying all results can be obtained directly from those public repositories. The project-generated summary tables and the analysis scripts are available from the corresponding authors on reasonable request.
